# Issue Analysis: A Use-Driven Approach to Data Governance Can Promote the Quality of Routine Health Data in India

**DOI:** 10.9745/GHSP-D-20-00347

**Published:** 2021-06-30

**Authors:** Nidhi Khurana

**Affiliations:** a Population Council, New Delhi, India.

## Abstract

India lacks a functional public policy framework to guide health data use and sharing practices, which stymies data quality. Embedding data governance in health data systems can promote quality and make service delivery more efficient. Much of the discourse on health information systems has focused on technology while the concern of using data for health system management and improving quality of care remains largely unaddressed.

## Teaser

**Key Messages:**

Key issues in India's health information systems include:

- Myriad data systems collect reams of public health data, often much more than necessary.
- These data systems function in silos and do not lend themselves to a holistic view of the health system's performance.
- India lacks a functional public policy framework to guide health data use and sharing, and the data systems are often not interoperable.

**Key Implications:**

Key considerations for policymakers:

- Deliberate and agree upon a robust health data sharing and use policy framework that enables user-centric data flows and inter-system data sharing.
- Make actionability the bedrock of data collection efforts by ensuring that systems collect data that either contribute to indicators for measuring health systems performance or support health workers in critical daily tasks.
- Democratize access to aggregate public health data to citizens and researchers. Set up institutional mechanisms for use of data for research to safeguard citizens' privacy rights.
- Embed true interoperability, prioritize decentralization, and consider legacy systems in health information system design.
- Engage with the private sector to collect data to create a more comprehensive picture of health care service delivery and quality in the country.

## MULTIPLE SYSTEMS FOR COLLECTING PUBLIC HEALTH DATA IN INDIA INTRODUCE AVOIDABLE REDUNDANCIES

The health data ecosystem in India consists of several parallel systems for collecting public health data, including the health management information system (HMIS), the Mother and Child Tracking System/Reproductive and Child Health Portal (MCTS/RCH), and other state-specific health data monitoring systems.^1^^,^^2^

The Ministry of Health and Family Welfare (MOHFW) created the HMIS to monitor health programs and provide key inputs for policy formulation and interventions. Currently, around 200,000 health facilities across all districts in India upload facility data every month directly on the HMIS web portal.^3^ Launched in 2009,^1^ the MCTS captured information on the delivery of the full spectrum of health care and immunization services to pregnant women and children aged up to 5 years. It tracks individual beneficiaries, as opposed to the older HMIS (first rolled out in 2005 with an upgrade in 2008), which captures service delivery information at an aggregate level.^2^ The MCTS, which focuses only on maternal and child health, was created due to reported gaps in the HMIS, which records data across health programs including reproductive health.^4^ There are several other state-level initiatives on reproductive health data that have either been subsumed under or run parallel to the MCTS.^4^

Since 2015, MCTS is gradually being phased out and replaced by the RCH portal.^5^ RCH, an upgraded version of MCTS, was designed for early identification and tracking of the individual beneficiary throughout the reproductive lifecycle.^3^^,^^5^^,^^6^ The RCH portal aims to track eligible couples for their contraceptive needs,^4^ in addition to monitoring service delivery for pregnant women and children. This is more comprehensive compared to the MCTS, which restricts data gathering to antenatal and postnatal periods and deliveries.^4^ The MOHFW has introduced a tablet-based application, called ANMOL (Auxiliary Nurse Midwife Online) for the RCH portal. It enables auxiliary nurse-midwives (ANMs) to enter data for beneficiaries of their jurisdiction, improving the data quality as the data are entered “at source” by health service providers.^4^

In addition, the Integrated Disease Surveillance Programme (IDSP), Routine Immunization Programme, and vertical disease programs, such as those for malaria and TB, also collect health data.^7^

Because India lacks a functional public policy framework to guide health data use and sharing practices, these data systems often function in silos with little interoperability^7^ Most of these systems, including the HMIS, are expensive, proprietary, and inflexible,^7^ despite the government's policy on the use of open-source software for public data systems.^8^^,^^9^

A key tenet of ensuring data quality and reducing the burden on health workers is that no data should be collected more than once. However, the existing data systems are riddled with redundancies on the one hand and important gaps on the other. For example, childhood TB data are collected under HMIS, IDSP, and Expanded Program for Immunization, with obvious duplication and adverse impacts on data quality and its use. Differing estimates of prevalence and coverage are likely to be a point of confusion for decision makers and program implementers, who may wonder about the reliability of these and therefore avoid using these data for health systems planning or program management. While so much redundant data are collected, there are important gaps, such as the lack of data on antimicrobial resistance, that make it difficult to assess the burden and programs. Also, the MCTS/RCH portal only collects quantitative data, while missing aspects such as community engagement, which is equally important for health services provision. Community engagement, which is a key marker of trust in the health system, can be determined by health service utilization trends and through periodic surveys on service provision quality and patient satisfaction at health facilities.

The mainstream discourse on health information systems has focused on the technology and the potential of standardized health and demographic data for artificial intelligence applications. Although there have been efforts to improve data quality and use for informing policy and programs, the topical concern of using data for health system management and improving quality of care can be addressed more effectively.^10^^,^^11^ Importantly, high-quality data are a prerequisite for robust data models to develop artificial intelligence applications for health systems.^12^

This highlights several important issues.

### 1. Actionable Health Data Is Still Hard to Come By

The use of data to inform policies and programs to improve health service delivery and health outcomes is not commensurate with the huge public investments in data collection. This is a problem of design for the symbolism of reporting rather than use for driving responsive health system management. Further, a failure to recognize the importance of engaging users of data at all levels to find out what they need also contributes to this poor data use culture. A study assessed that the overall data collection volume for aggregate data (HMIS data and state-specific data) varies between 3,000 to 8,000 data elements per month.^7^ Further, only 10% of these data elements are used to generate indicators, nearly 20% were inactive (returned no values), and about 50% consistently returned blanks or zeroes.^7^ This clearly shows that the systems collect far more data than what's reported on and an even smaller proportion is fed into meaningful indicators. The zero/blank value elements may point to gaps in a facility's readiness to deliver certain services (e.g., if “zero” stunted children are reported consistently by a given facility in a poor district, it is more likely that the facility doesn't have weighing scales and height measures rather than that there are no stunted children). Currently, when a “zero/blank” is reported for a given service at a health facility, there's no way to tell if it represents a nonutilized service or a nonexistent service. One way to circumvent this could be to make all data fields required when submitting electronic data (so they cannot be left blank) and to add a pop-up list of options for elaboration. The list could include service not offered, no clients, equipment/medicine shortage, inadequate staffing, other reason, so that appropriate programmatic action may be taken. Additionally, if facility staff are entering data on a paper-based form, provide space for recording a short list of options, to make it easier for staff to complete the form and for whoever is eventually entering the form into an electronic database.

Systems collect far more data than what's reported on and an even smaller proportion is fed into meaningful indicators.

Furthermore, for public health data, denominators are crucial.^7^ Although absolute numbers (e.g., number of children immunized) are readily available, it is often difficult to ascertain coverage levels of health services such as the percentage of children immunized fully (immunization coverage) because the denominators remain disparate and vary widely across administrative levels, departments, and health programs. The coverage indicators are vitally important from a health systems performance management perspective, as they reflect the extent to which the people in need receive important health interventions. At times, the absence of standardized denominators creates confusion in setting targets for health facilities due to a lack of a common understanding of how much ground has been covered between different levels or programs.

Additionally, since data quality functions are largely centralized—meaning that there is no standardized approach to checking data at the local levels—the principle of “data quality corrections are best done closest to the source of data collection” is violated.

### 2. Private Health Care Sector Generates a Lot of Data but Barely Reports Any

The National Sample Survey 2014 estimated that of all episodes of illness, 72% of rural episodes and 79% of urban, were treated in the private sector. The private sector provides about two-thirds of inpatient care and three-fourths of outpatient care treatments in India. It has been estimated that 60% of the total volume of health data is produced by non-state actors.^13^ However, in the absence of regulation and incentives, the reporting is minimal to nonexistent.

### 3. Citizens' Data Access and Privacy Needs to Be Addressed

Communities and researchers have limited access to routine administrative health data. Lack of data sharing can affect the people's trust in the system as they do not know what services are provided or their quality. This, in turn, has implications for citizens' empowerment and mobilization for better population health because they don't have information on the major causes of illness and death and what services are underutilized, apart from stymieing data used for research to improve the quality of care and health outcomes.

Lack of adequate data regulation and privacy standards while collecting case-based data makes households vulnerable to unsolicited phone calls from call centers to verify a pregnancy and potential data mining for market research, apart from graver implications such as stigma or discrimination against people with certain health conditions. Further, data are only as good as the trust that people have in the confidentiality of the data. Reports of misuse or breach of privacy (e.g., through the media) could impact future response rates, make respondents likely to withhold or falsify information, or withdraw their consent to share the data, thereby impacting the quality of the data. Health data systems can therefore benefit from an inclusive, learning, and iterative data governance style, which allows for decisions to be scrutinized and approaches to evolve, based on periodic inputs from the health workers, patients, and researchers.^14^^–^^16^

### 4. Reporting Data Into Multiple Systems Imposes Additional Administrative Burden on Health Workers

The MCTS/RCH portal sends all patient-level data to the national portal. However, most patient-level details, especially those pertaining to maternal and child health services, are only needed by the facility-level staff. The privacy risk and the sheer volume of patient-level data necessitate having a more selective system in which only necessary patient-level data are reported to the national level (e.g., data on outbreaks). Instead of using the current broad-brush approach to collecting data, more careful planning and assessment of what patient-level data are needed at the national level must be done and followed through. Further, parallel data reporting in MCTS/RCH portal and HMIS means that health workers have to enter the data twice.^7^

The government's introduction of and focus on the MCTS/RCH portal undermined the older HMIS by encumbering health workers who needed to focus on reporting into this new system. Furthermore, several other state-specific portals that were created to serve the needs of states and donors exacerbated their workload. While increasing the staff workload, who estimated that 60% of their time was spent on data-related work,^7^ the volume of data collected likely had implications for its quality. Such data of questionable quality does not lend itself to effective use while taking valuable health worker time away from their essential caregiving duties.^7^ According to a study, ANMs spent an average of 6 hours a week for data collection for MCTS in addition to their routine program activities.^17^ This issue has been addressed to some extent in the RCH by introducing ANMOL,^18^ designed in collaboration with United Nations Children's Fund. Data are collected at the source by health workers. ANMOL is being rolled out in phases across states and helps reduce the burden of manual data entry and travel for ANMs.

In HMIS, the redundancy in recording data introduces time lags in data collection and increases the risk of data errors.

However, in HMIS, there's redundancy in recording data because the ANMs first enter data in physical registers, which are then digitized by the data entry operators at the primary health centers. This step also introduces time lags in data collecting and reporting and increases the risk of data errors, especially if there's discordance between the terms used in the register columns and the software fields of the health data systems. Lack of HMIS data entry training for ANMs and the expectation that health workers enter data in the physical registers alone poses additional concerns about the data quality.^13^ Poor translation of medical terms, such as “eclampsia” and “hypothermia” in the local language, results in frequent misinterpretation and poor data quality.^13^ This is vastly different from the experience in some other developing countries, which have emphasized the need for greater participatory engagement with health workers and community members in designing the health information system before implementation to ensure sustainability.^4^^,^^19^

## INDIA'S RECENT DIGITAL HEALTH INITIATIVES AND PROPOSED LEGISLATIONS

The National Digital Health Mission (NDHM), launched in August 2020, is the Government of India's marquee program that envisions an integrated digital ecosystem for health care services in the country based on individual patient records. It aims to create a public digital infrastructure that empowers individuals, patients, doctors, health facilities and helps streamline the delivery of health care services and related information. With citizens at the center of the mission, the proposed digital ecosystem comprises diverse actors, including policy makers, health care providers, regulators, health care professionals, private insurers, health-technology companies, and nonprofit organizations.^20^

National Digital Health Mission is India's marquee program that envisions an integrated digital ecosystem for health care services.

The NDHM represents a stride forward from the National Digital Health Blueprint (NDHB) released in 2019, which recognized the need to establish the NDHM.^20^ The NDHB is an extension of the National Health Policy 2017, which espouses the use of digital technologies to provide universal health care. In 2018, a special committee was created to work on the blueprint.^20^ The NDHM includes 5 data systems as building blocks: (i) healthID, a unique patient identifier repository; (ii) DigiDoctor, a repository of all doctors enrolled in the country; (iii) Health Facility Registry, a repository of all health facilities in the country; (iv) NDHM Health Records, is an electronic record of a person's health-related information that conforms to nationally recognized interoperability standards and that can be drawn from multiple sources while being managed, shared, and controlled by the individual; and (v) Electronic Medical Records, a digital version of a patient's chart at a particular facility.^20^

It is important to emphasize that NDHM's success depends on its adoption by diverse stakeholders including the states, public and private providers, policy makers, program managers, and citizens. Although these registries have immense potential to provide data for decision making that can be used to inform health system planning and improvements, NDHM's implementation strategy needs to account for the fact that the public health sector is currently based largely on aggregated health information systems rather than patient-centric systems.^1^ Importantly, the guidance on navigating the transition from legacy systems to implementing patient-centric systems is thin.^7^ This could create more parallel systems where the new systems would impose additional burden on the providers who may lack the capacity to replace the older systems. Most private providers lack the incentive to register themselves because registration is “voluntary,” so they are likely to sidestep this to avoid procedural hassles or patient accountability. Under the central government's new hospital insurance scheme and other publicly funded insurance schemes, most private providers are not included. The private providers that are included, either report data only on a small subset of their patients or no data. NDHM's plans are redolent of challenges that India continues to face in implementing the Clinical Establishments (Registration and Regulation) Act, 2010 (CEA).^21^ Like the NDHM, CEA was promulgated by the central government and covers both public and private establishments such as single-doctor clinics, laboratories, and corporate hospitals.^22^ The establishments do not see the value of registering themselves under this act and want to sidestep malpractice lawsuits. More than 10 years after CEA was legislated, many states have not been able to implement it due to opposition from the private sector.^21^ There have been multiple instances of doctors, supported by the Indian Medical Association, going on strike to protest the implementation of this act.^23^^–^^25^

NDHM's success depends on its adoption by diverse stakeholders.

For policy implementation, sequence matters. The Indian government has 2 data protection bills in the works that offer mechanisms to assure data privacy for citizens and establish data governance mechanisms^26^: the Digital Information Security in Healthcare Act (DISHA) and the Personal Data Protection Bill. DISHA's provisions include seeking informed consent and the right to refuse data sharing, protecting against commercial usage of digital health data, and permitting patients to complete their incomplete data or rectify inaccurate information.^27^ The data protection bill addresses data governance by laying out the responsibilities of data fiduciaries (data collectors) and the rights of data principals (those about whom data are being collected).^28^ It might be prudent to implement the NDHM once these laws are passed so that citizens' privacy rights are legally assured. Waiting would have the added benefit of circumventing the need for post-facto amendments to NDHM per the finally legislated data governance frameworks and would likely help drive better adoption of the NDHM by the citizens and help engender public trust in these new data systems.

## CALL TO ACTION: WHAT MIGHT WE DO TO STRENGTHEN HEALTH DATA GOVERNANCE?

Institutionalizing data governance by establishing a shared, cogent framework in health data systems at all levels of policy and practice, alongside a user-centric design of workflows can promote quality, protect privacy, boost innovative research, and enable service delivery efficiencies. The foundational principle of a robust national health data governance framework is that high-quality data are available to address the information needs of decision makers while protecting citizen's privacy rights and minimizing the burden on health care workers.

### 1. Make Data Collection About Use and Actionability

It is vital that the systems collect data that either contribute to indicators or support health workers in critical daily tasks. The guiding principle should be to reduce the health care workers' burden while strengthening health systems. The decisions about which data elements to capture should begin with what is needed to support the daily activities of the frontline health workers, see what can be repurposed for computing aggregate indicators, and only then, consider additional data elements for collection. The digital tracking and support systems should be user-centered and emphasize the principle of "collect once, use for many purposes"—so that data collected for service delivery can also be used for accountability (i.e., to calculate aggregate indicators required for reporting and monitoring provider, stock, and system performance).

The guiding principle of data collection should be to reduce the health care workers' burden while strengthening health systems.

The national health program should specify the information that it needs for public health management and policy and how often and allow state health departments to deliver this information the best way they can. Reducing the volume of data would make it easier for states to validate data and ensure data quality. Further, data may be fragmented across the ministry, state, and agency silos. A lack of data standards for reporting and interoperability between data systems can limit the ability to synthesize information across multiple data sources to fully understand programmatic issues.^29^ An open dialogue between producers and consumers of data at both the national and subnational levels on the various data elements, duplications, and gaps, can help harmonize data sharing and use and improve data quality.

### 2. Enable Health Workers to Enter Accurate Data Directly in HMIS

To eliminate the issues of duplicative work, time lag between collection and reporting, discordance between paper and digital records, and poor translation, the health workers should be supported to enter data directly and accurately at the point of care. Apart from organizing data entry training on HMIS, every effort should be made to include clear translations of medical terms in the local language.

### 3. Make Decentralization a Priority

The main purpose of information technology systems in the states and districts should be for decentralized management at that level. The electronic health records should typically be maintained at the facility level and only aggregate data stored in the cloud. No level should receive routine data for more than 2 levels below (which implies no patient data should go above block or district, other than what's necessary from a high-level policy standpoint, such as data on outbreaks). This will keep the systems decentralized and ensure that the center sees only what it needs for policy or programmatic action. Mature public health systems may provide patient-specific data from each facility with considerable granularity whereas less mature systems can give aggregate numbers from a block or district level. Decentralization addresses multiple challenges at once: helping maintain citizens' data privacy and focusing on what's actionable at each level of the health system while obviating the need for multiple systems.

### 4. Embed True Interoperability

Lack of interoperability precludes a unified view of multiple data sources to comprehensively understand programmatic issues and use this to monitor and improve health systems performance.^29^ Despite government policies on the use of open-source software for all public systems, the Application Program Interface (API) access for the national HMIS (which is built on a proprietary platform) is not currently available to the states. For instance, in 2012, the National Data Sharing and Accessibility Policy was announced to strengthen sharing of information across ministries and systems to promote evidence-based decision making, while discouraging data duplications.^30^ In 2015, the government issued its open API policy, which recognizes the need for an interoperable data ecosystem, applications, and processes to make the right information available to the right user at the right times.^31^ So, it might be worthwhile for the government to do a stock-taking exercise to assess whether the code is available on public repositories and if open API access is provided.

### 5. Consider Legacy Systems and Transition

While introducing new systems, it's imperative to be deliberate and judicious. A new system may not always fix existing problems and can create new problems because it may not be appropriate for all technical areas nor be interoperable with other systems. So, while new systems are being proposed to replace all others (e.g., the Integrated Health Information Portal [IHIP], which began as a system for IDSP is now looking to include 25 other programs), it may not be feasible to incorporate the needs of different health programs into 1 software. Further, while introducing new systems, the legacy data systems landscape should be considered to enable a smooth transition. For example, the efforts of the National Vector Borne Diseases Control Program to develop a malaria surveillance system were put on hold as the IHIP was supposed to address it, but as the operationalization of IHIP has been delayed,^7^ this has resulted in an avoidable data gap.

A new data system may not always fix existing problems and can create new problems.

Finally, the World Health Organization has developed standard modules for TB, malaria, Expanded Program for Immunization, HIV, and mortality reporting on the open-source District Health Information Software-2 (DHIS2) platform and has invited countries to adapt these. So, there's also the question of whether India should seek to work on these globally designed standards as opposed to having new systems developed by information technology vendors who may have a limited understanding of public health. The focus should likely be on data integration and interoperability instead of software integration.

### 6. Seek to Engage Private Health Care Providers to Gather Data

As most providers are not included under the central government's new hospital insurance scheme, they need to be connected to portals to provide the required aggregate information. Further, they may not be willing to provide patient-specific information and may have to be engaged in dialogue to better understand their concerns and assure them on anonymizing the collected data.

### 7. Define Institutional Mechanisms for Using Data for Research

Separate mechanisms and authorities must be created to use data from central repositories for research to verify the aims of third parties wishing to access data so that data are only used appropriately for research. Research use must not be conflated with the use of personal health identifiers in individual patient care. This is important to prevent data mining for market research as opposed to academic research to keep the sanctity of the purpose for which the individuals trusted providers with the data.

### 8. Make Aggregate Data Widely Available

Data collected by routine health information systems, government programs, and large-scale surveys for policy purposes, as well as the data collected by health and biomedical research institutions using public funds, should be made publicly available for researchers and advocates, while ensuring that it is not used for market research. Making data widely available builds the citizens' trust in public data and enables responsive governance through generating high-quality data.

## CONCLUSION

Data quality and data use constitute a virtuous cycle. Data quality can be compromised due to burdensome data collection processes at local service delivery levels, due to complex reporting procedures (e.g., multiple reporting forms), as well as a lack of standardized and harmonized systems for data collection.^32^ A considered and shared framework to guide the use and sharing of public health data can help streamline data flows and enable intersystem data sharing. This is likely to promote the use of existing data for policy making and planning by ensuring that data systems generate relevant indicators for measuring health systems performance. A large volume of data of suspect quality does not lend itself to effective use. High-quality data are more likely to be used, and higher use for research and policy making in turn engenders efforts to improve data quality by reviewing the data flows and analyses and sparking conversations on appropriate indicators and denominators. This virtuous cycle of data quality and data use for decision making lends itself to strengthening health systems performance and improving the quality of service provision while also protecting privacy and building research use.

## Notes

**Peer Reviewed**
